# Kinetics of Micellization and Liquid–Liquid Phase Separation in Dilute Block Copolymer Solutions

**DOI:** 10.3390/polym15030708

**Published:** 2023-01-31

**Authors:** Takahiro Sato

**Affiliations:** Osaka Study Center, The Open University of Japan, 4-9-23, Onohara-Higashi, Osaka 562-0031, Japan; tsato@chem.sci.osaka-u.ac.jp; Tel.: +81-72-730-8410

**Keywords:** block copolymer, micelle, concentrated-phase droplet, phase separation kinetics, lattice theory

## Abstract

A lattice theory for block copolymer solutions near the boundary between the micellization and liquid–liquid phase separation regions proposes a new kinetic process of micellization where small concentrated-phase droplets are first formed and then transformed into micelles in the early stage of micellization. Moreover, the thermodynamically stable concentrated phase formed from metastable micelles by a unique ripening process in the late stage of phase separation, where the growing concentrated-phase droplet size is proportional to the square root of the time.

## 1. Introduction

An amphiphilic diblock copolymer forms a micelle in a selective solvent. If the block copolymer is thermosensitive, pH-sensitive, or ionic strength-sensitive, the kinetics of the micelle formation in dilute homogeneous block copolymer solutions can be studied experimentally by temperature-jump, pH-jump, or salt-jump experiments. Such kinetic studies have been carried out extensively for various block copolymer solutions thus far [1,2,3,4,5,6,7,8,9,10,11,12,13,14,15,16]. The micellization process of block copolymers is much slower than that of small molecular surfactants, so the former kinetics is easier than the latter, but the micellization of the block copolymers is still fast to study experimentally.

The micellization kinetics of both block copolymers and small molecular surfactants have been analyzed on the basis of the stepwise aggregation mechanism of monomers [17,18,19,20,21]. This mechanism was first proposed by Aniansson and Wall in 1974 [17] to explain the micellization kinetics of small molecular surfactants, which resembles the kinetic theory of nucleation (the formation of a new phase within a metastable phase) [19,21], and was also utilized to analyze block copolymer micellization kinetics [3,4,5,6].

More recently, our group investigated the self-assemblies of several thermosensitive and ionic strength-sensitive block copolymers in dilute aqueous solutions, and found the competition between the micellization and liquid–liquid phase separation of those block copolymers [22,23,24,25,26]. The competition comes from weaker amphiphilicity of the block copolymers, which was explained semi-quantitatively by the lattice theory [27]. Furthermore, the lattice theory predicted that micellization prefers to liquid–liquid phase separation at a lower hydrophobic content of the copolymers, and the prediction was demonstrated experimentally using a thermosensitive block copolymer in aqueous solutions [26].

The present study deals with the kinetics of the micellization as well as of the liquid–liquid phase separation in dilute block copolymer solutions, where the micellization competes with the liquid–liquid phase separation. The liquid–liquid phase separation starts from the formation of small concentrated-phase droplets, which are thermodynamically less stable than the macroscopic concentrated phase due to the extra interfacial Gibbs energy. The preference of concentrated-phase droplets and micelles may be reversed during the growing process of micellization or phase separation. The judgement of the preference needs to quantitatively compare the Gibbs energy of formation of the concentrated-phase droplet with that of the micelle. Such a comparison proposes the unique kinetics of micellization and concentrated-phase droplet growth in the early and late stage, respectively, as explained in what follows.

## 2. Thermodynamics

### 2.1. Models and Mixing Gibbs Energy Densities [27]

Let us consider an aqueous solution of a block copolymer that consists of a hydrophilic A-chain of the degree of polymerization *P*_A_ and a hydrophobic B-chain of the degree of polymerization *P*_B_. The hydrophilic and hydrophobic contents of the copolymer are given by *x*_A_ = *P*_A_/(*P*_A_ + *P*_B_) and *x*_B_ = *P*_B_/(*P*_A_ + *P*_B_), respectively. The monomer units of the A- and B-chains as well as the solvent water molecule are assumed to possess the same size *a* (the unit lattice size).

**Figure 1:**
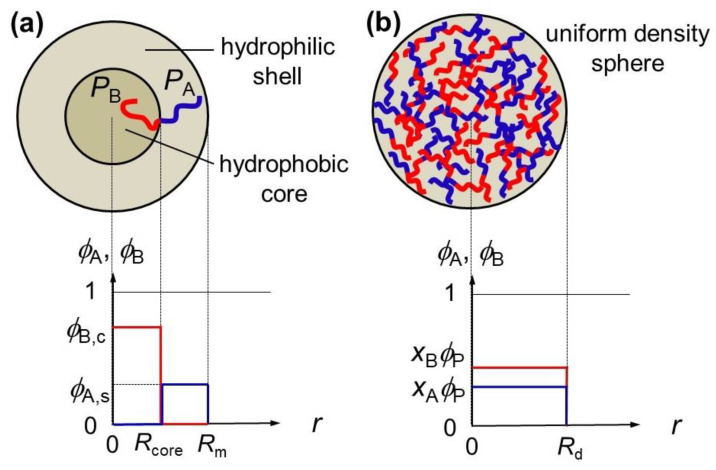
Simplified model of the spherical micelle (**a**) and the uniform density spherical model of the concentrated-phase droplet (**b**).

Figure 1a shows a schematic illustration of the spherical micelle. We assumed that both the hydrophobic core and hydrophilic shell regions of the micelle were uniform, and that the A- and B-block chains were completely excluded from the core and shell regions of the micelle, respectively, as shown in Figure 1, to calculate the thermodynamic quantities of the micellar phase. In this theory, the micelle is regarded as a thermodynamic phase. This simplified model of the spherical micelle resembles that used by Leibler et al. [28], although in the present model, the radii of the core *R*_core_ and of the whole micelle *R*_m_ are given by

(1) $$R_{\mathrm{core}}/a=P_{B}{}^{\alpha },\;\left(R_{m}-R_{\mathrm{core}}\right)/a=P_{A}{}^{\alpha }$$

Here, the exponent *α* was assumed to be 0.5 for the Gaussian chain in this paper, but this *α* value does not essentially change the following semi-quantitative argument. Leibler et al. [28] considered a homopolymer as the solvent, and treated *R*_core_ and *R*_m_ as variables, determined from the free energy minimization condition.

The average volume fraction of the copolymer *ϕ*_P_ in the micellar phase is given by *ϕ*_P_ = 3(*P*_A_ + *P*_B_)*m*/(4*πR*_m_^3^), where *m* is the aggregation number of copolymer chains in the spherical micelle. The volume fractions of the A- and B-block chains in the shell and core regions (*ϕ*_A,s_ and *ϕ*_B,c_), respectively, can be calculated from *ϕ*_P_ by

(2) $$\phi_{A,s}=\frac{x_{A}\phi_{P}}{\Phi_{s}},\phi_{B,c}=\frac{x_{B}\phi_{P}}{\Phi_{c}},\Phi_{c}=1-\Phi_{s}\equiv \frac{R_{\mathrm{core}}{}^{3}}{R_{m}{}^{3}}$$

By extending the Flory–Huggins theory [29], we can formulate the mixing Gibbs energy density (per the unit lattice site) ∆*g*_m_(*ϕ*_P_) in the micellar phase as [27]

(3) $$\begin{array}{c} \frac{\Delta g_{m}(\phi_{P})}{k_{B}T}=\frac{\phi_{P}}{P}\ln\left(\kappa \phi_{P}\right)+\Phi_{s}\left(1-\phi_{A,s}\right)\ln\left(1-\phi_{A,s}\right)+\Phi_{c}\left(1-\phi_{B,c}\right)\ln\left(1-\phi_{B,c}\right)\\ +\left[x_{A}\left(1-\phi_{A,s}\right)\chi_{\mathrm{AS}}+x_{B}\left(1-\phi_{B,c}\right)\chi_{\mathrm{BS}}-x_{A}x_{B}\chi_{\mathrm{AB}}\right]\phi_{P}+\frac{3\Phi_{c}}{R_{\mathrm{core}}/a}\frac{a^{2}\gamma_{c}}{k_{B}T} \end{array}$$

where *k*_B_*T* is the Boltzmann constant multiplied by the absolute temperature, and *κ* is a constant defined by

(4) $$\kappa =\frac{\pi }{27}{\left(P_{A}+P_{B}\right)}^{2+\alpha }x_{A}x_{B}{}^{1-2\alpha }{\left(x_{A}{}^{\alpha }+x_{B}{}^{\alpha }\right)}^{3}$$

**Figure A1:**
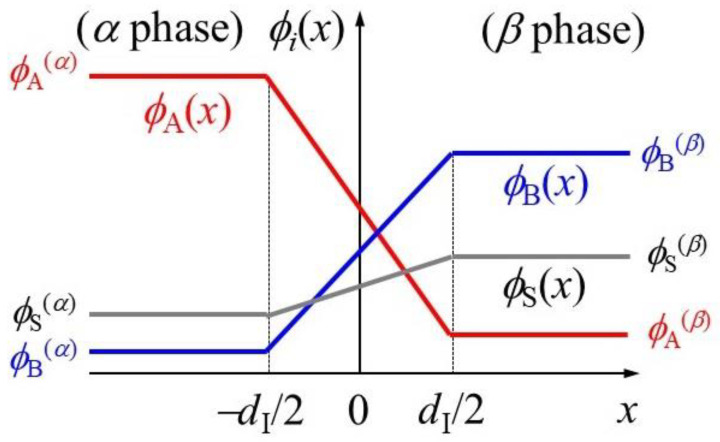
Concentration distribution of homopolymers A and B and the solvent near the interfacial region in the phase-separating ternary solution.

*χ*_AS_, *χ*_BS_, and *χ*_AB_ are the interaction parameters between the A-chain and the solvent, between the B-chain and the solvent, and between the A- and B-chains, respectively, and *γ*_c_ is the interfacial tension between the core and shell regions. Using the theory of Noolandi and Hong [30], as explained in Appendix A, the expression of *γ*_c_ as well as the thicknesses of the corresponding interfaces *d*_I,c_ are given by

(5) $$\frac{a^{2}\gamma_{c}}{k_{B}T}=\sqrt{\frac{\left(\phi_{A,s}+\phi_{B,c}\right)\Delta f_{c}(0)}{3}},\frac{d_{I,c}}{a}=\sqrt{\frac{\phi_{A,s}+\phi_{B,c}}{12\Delta f_{c}(0)}}$$

where ∆*f*_c_(0) is defined as

(6) $$\begin{array}{c} 4\Delta f_{c}(0)\equiv \chi_{\mathrm{AB}}\phi_{A,s}\phi_{B,c}+\left(\chi_{\mathrm{BS}}\phi_{B,c}-\chi_{\mathrm{AS}}\phi_{A,s}\right)\left(\phi_{B,c}-\phi_{A,s}\right)\\ +2\phi_{S,c}\ln\left(\frac{\phi_{S,c}+\phi_{S,s}}{2\phi_{S,c}}\right)+2\phi_{S,s}\ln\left(\frac{\phi_{S,c}+\phi_{S,s}}{2\phi_{S,s}}\right) \end{array}$$

with *ϕ*_S,s_ = 1 − *ϕ*_A,s_ and *ϕ*_S,c_ = 1 − *ϕ*_B,s_. Although not shown in Figure 1a, the interfacial region of the micelle has a sharp concentration gradient, as illustrated in Figure A1 in Appendix A. In Equation (3), the term of the interfacial tension between the shell region of the micelle and the coexisting dilute phase was neglected, because *ϕ*_A,s_ and *χ*_AS_ were considerably smaller than *ϕ*_P,d_ and $\bar{\chi }$ (cf. Equation (8)), respectively.

Figure 1b illustrates the schematic diagram of the spherical concentrated-phase droplet, where both volume fractions of the A- and B-chains are uniform. Through a simple extension of the Flory–Huggins theory [29], the mixing Gibbs energy density ∆*g*_d_(*ϕ*_P_) in the concentrated-phase droplet is written as

(7) $$\frac{\Delta g_{d}(\phi_{P})}{k_{B}T}=\phi_{S}\ln\phi_{S}+\frac{\phi_{P}}{P}\ln\phi_{P}+\bar{\chi }\phi_{S}\phi_{P}+\frac{3a}{R_{d}}\frac{a^{2}\gamma_{d}}{k_{B}T}$$

where *ϕ*_P_ and *ϕ*_S_ = 1 − *ϕ*_P_ are the volume fractions of the copolymer and the solvent in the droplet phase, respectively, and $\bar{\chi }$ is the average interaction parameter defined by

(8) $$\bar{\chi }\equiv \chi_{\mathrm{AS}}x_{A}+\chi_{\mathrm{BS}}x_{B}-\chi_{\mathrm{AB}}x_{A}x_{B}$$

and *γ*_d_ is the interfacial tension between the concentrated-phase droplet and the homogeneous (dilute) solution phase. Not illustrated in Figure 1b, the interfacial region of the droplet must possess a sharp concentration gradient. There are two limiting cases: one is that only A-block chains make contact with the coexisting dilute phase at the interface, like the micelle, and the other is to assume that the composition *ϕ*_A_(*x*)/*ϕ*_B_(*x*) (cf. Figure A1 in Appendix A) is uniform, even at the interface. Here, we took the latter limiting case (i.e., the total copolymer volume fraction *ϕ*_P_(*x*) changes under the constant composition). Then, the expression of *γ*_d_ and the thickness of this interface *d*_I,d_ are obtained from Equations (A4)–(A6) in Appendix A by

(9) $$\frac{a^{2}\gamma_{d}}{k_{B}T}=\sqrt{\frac{\Delta f_{d}(0)}{3\left[\phi_{P}+\phi_{P,h}(R_{d})\right]}}\left[\phi_{P}-\phi_{P,h}(R_{d})\right],\frac{d_{I,d}}{a}=\frac{\phi_{P}-\phi_{P,h}(R_{d})}{\sqrt{12\left[\phi_{P}+\phi_{P,h}(R_{d})\right]\Delta f_{d}(0)}}$$

with the copolymer volume fraction *ϕ*_P,h_(*R*_d_) in the dilute solution phase coexisting with the concentrated-phase droplet of the radius *R*_d_ and

(10) $$\begin{array}{c} 4\Delta f_{d}(0)\equiv \bar{\chi }\left[\phi_{P}-\phi_{P,h}(R_{d})\right]\left[\phi_{S,h}(R_{d})-\phi_{S}\right]\\ \;\;\;\;\;\;\;\;+2\phi_{S}\ln\left[\frac{\phi_{S}+\phi_{S,h}(R_{d})}{2\phi_{S}}\right]+2\phi_{S,h}(R_{d})\ln\left[\frac{\phi_{S}+\phi_{S,h}(R_{d})}{2\phi_{S,h}(R_{d})}\right] \end{array}$$

[*ϕ*_S_ = 1 − *ϕ*_P_ and *ϕ*_S,h_(*R*_d_) = 1 − *ϕ*_P,h_(*R*_d_)].

The excess chemical potentials (per molecule) of the solvent ∆*μ*_S,d_ and of the copolymer ∆*μ*_P,d_ in the droplet phase can be calculated from Equation (7) as

(11) $$\begin{array}{c} \frac{\Delta \mu_{S,d}}{k_{B}T}=\ln\phi_{S}+\left(1-\frac{1}{P}\right)\phi_{P}+\bar{\chi }\phi_{P}{}^{2}+\frac{2}{\left(R_{d}/a\right)}\frac{a^{2}\gamma_{d}}{k_{B}T}-\frac{3\phi_{P}}{\left(R_{d}/a\right)}\left(\frac{d}{d\phi_{P}}\frac{a^{2}\gamma_{d}}{k_{B}T}\right)\\ \frac{\Delta \mu_{P,d}/P}{k_{B}T}=\frac{1}{P}\ln\phi_{P}-\left(1-\frac{1}{P}\right)\phi_{S}+\bar{\chi }\phi_{S}{}^{2}+\frac{2}{\left(R_{d}/a\right)}\frac{a^{2}\gamma_{d}}{k_{B}T}+\frac{3\phi_{S}}{\left(R_{d}/a\right)}\left(\frac{d}{d\phi_{P}}\frac{a^{2}\gamma_{d}}{k_{B}T}\right) \end{array}$$

Finally, the mixing Gibbs energy density ∆*g*_h_(*ϕ*_P_) as well as the excess chemical potentials of the solvent ∆*μ*_S,h_ and of the copolymer ∆*μ*_P,h_ in the homogeneous copolymer solution phase are given by [29]

(12) $$\begin{array}{c} \frac{\Delta g_{h}(\phi_{P})}{k_{B}T}=\phi_{S}\ln\phi_{S}+\frac{\phi_{P}}{P}\ln\phi_{P}+\bar{\chi }\phi_{S}\phi_{P}\\ \frac{\Delta \mu_{S,h}}{k_{B}T}=\ln\phi_{S}+\left(1-\frac{1}{P}\right)\phi_{P}+\bar{\chi }\phi_{P}{}^{2},\frac{\Delta \mu_{P,h}/P}{k_{B}T}=\frac{1}{P}\ln\phi_{P}-\left(1-\frac{1}{P}\right)\phi_{S}+\bar{\chi }\phi_{S}{}^{2} \end{array}$$

which correspond to Equations (7) and (11) in the limit of infinite *R*_d_.

### 2.2. Phase Diagram

When $\bar{\chi }$ is sufficiently large, concentrated-phase droplets with the copolymer volume fraction *ϕ*_P,d_(*R*_d_) appear in the homogeneous copolymer solution, and if the amphiphilicity is strong enough (or *χ*_BS_ >> *χ*_AS_), the micellar phase with the copolymer volume fraction *ϕ*_P,m_ is formed in the homogeneous copolymer solution. The copolymer volume fractions *ϕ*_P,d_(*R*_d_) and *ϕ*_P,m_ can be calculated from the following phase equilibrium conditions

(13a) $$\Delta \mu_{S,h}(\phi_{P,h}(R_{d}))=\Delta \mu_{S,d}(\phi_{P,d}(R_{d})),\Delta \mu_{P,h}(\phi_{P,h}(R_{d}))=\Delta \mu_{P,d}(\phi_{P,d}(R_{d}))$$

(13b) $$\Delta \mu_{S,h}(\phi_{P,h}{}^{\left(m\right)})=\Delta \mu_{S,m}(\phi_{P,m}),\Delta \mu_{P,h}(\phi_{P,h}{}^{\left(m\right)})=\Delta \mu_{P,m}(\phi_{P,m})$$

where ∆*μ*_S,*I*_ and ∆*μ*_P,*i*_ (*i* = h, d, m) are the excess chemical potentials (per molecule) of the solvent and of the copolymer, respectively, in the homogeneous solution (*i* = h), in the droplet phase (*i* = d), and in the micellar phase (*i* = m); *ϕ*_P,h_(*R*_d_) and *ϕ*_P,h_^(m)^ are the copolymer volume fractions of the homogeneous (dilute) phase coexisting with the droplet and micellar phases, respectively.

**Figure 2:**
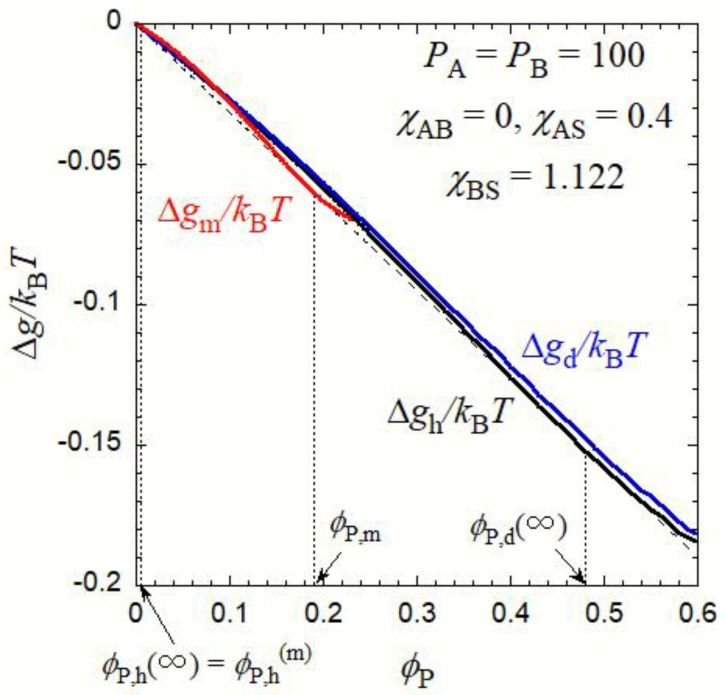
Mixing Gibbs energy densities, ∆*g*_h_ (the black solid curve), ∆*g*_d_ (the blue solid curve), and ∆*g*_m_ (the red solid curve) as functions of *ϕ*_P_ for an AB diblock copolymer solution with *P*_A_ = *P*_B_ = 100 (*x*_A_ = *x*_B_ = 0.5), *χ*_AS_ = 0.4, *χ*_AB_ = 0, and *χ*_BS_ = 1.122. The thin dashed line indicates the common tangent.

As a case study, Figure 2 shows three mixing Gibbs energy densities, ∆*g*_h_, ∆*g*_d_, and ∆*g*_m_ as functions of *ϕ*_P_ for an AB diblock copolymer solution with *P*_A_ = *P*_B_ = 100 (*x*_A_ = *x*_B_ = 0.5), *χ*_AS_ = 0.4, *χ*_AB_ = 0, and *χ*_BS_ = 1.122. The blue curve (∆*g*_d_) for the droplet phase shifts slightly upward from the black curve (∆*g*_h_) for the homogeneous solution due to the interfacial tension term in Equation (7). The red curve (∆*g*_m_) for the micellar phase has stronger curvature than the black and blue curves. By choosing *χ*_BS_ = 1.122, we can draw a common tangent to the black and red curves (although not clearly shown, the black curve was convex downward at *ϕ*_P_~0), which makes contact with the black and red curves at *ϕ*_P,h_(∞) (=*ϕ*_P,h_^(m)^), *ϕ*_P,m_, and *ϕ*_P,d_(∞). When *χ*_BS_ < 1.122, the red curve shifts upward, and when *χ*_BS_ > 1.122, the red curve shifts downward relative to the black curve, indicating that the homogeneous solution and the micellar phase are thermodynamically stabler at lower and higher *χ*_BS_, respectively [27].

From the Gibbs–Duhem relation, the mixing Gibbs energy density is related to the excess chemical potential by

(14) $$\Delta g_{h}(\phi_{P})=\left(1-\phi_{P}\right)\Delta \mu_{S,h}+\phi_{P}\frac{\Delta \mu_{P,h}}{P},\Delta g_{m}(\phi_{P})=\left(1-\phi_{P}\right)\Delta \mu_{S,m}+\phi_{P}\frac{\Delta \mu_{P,m}}{P}$$

and this equation verifies that *ϕ*_P,h_^(m)^ and *ϕ*_P,m_ in Figure 2 fulfill the phase equilibrium conditions given by Equation (13b). On the other hand, the Gibbs–Duhem relation does not hold for the droplet phase due to the interfacial tension term in Equation (7), which is not proportional to the total number of molecules in the system. However, the coexisting phase volume fractions *ϕ*_P,d_(*R*_d_) and *ϕ*_P,h_(*R*_d_) can be calculated by solving the simultaneous Equation (13a).

**Figure 3:**
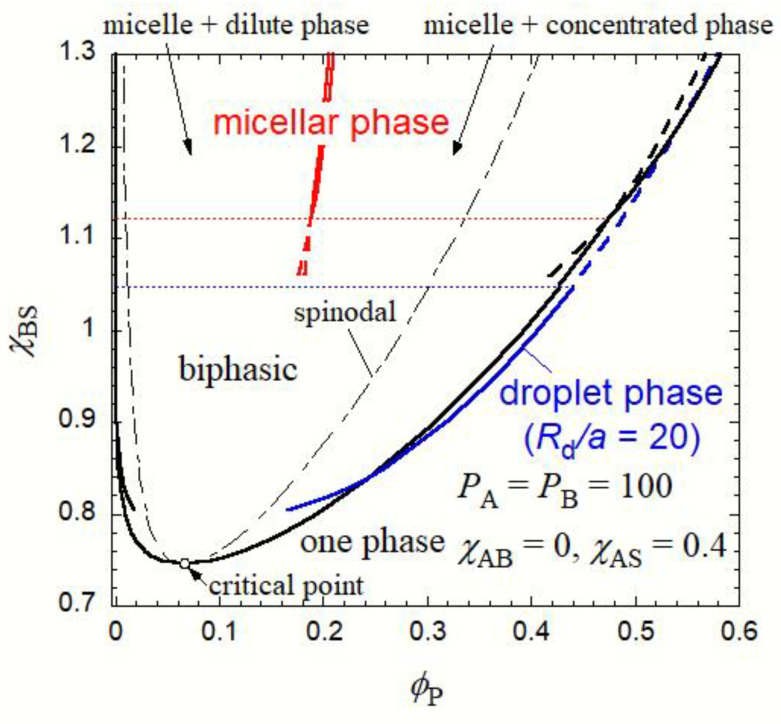
Phase diagram of the AB diblock copolymer solution with *P*_A_ = *P*_B_ = 100 (*x*_A_ = *x*_B_ = 0.5), *χ*_AS_ = 0.4 and *χ*_AB_ = 0.

Pairs of the coexisting phase volume fractions, *ϕ*_P,h_(∞) and *ϕ*_P,d_(∞), *ϕ*_P,d_(*R*_d_) and *ϕ*_P,h_(*R*_d_), and *ϕ*_P,h_^(m)^ and *ϕ*_P,m_, are obtained as functions of *χ*_BS_, at constant *χ*_AS_ = 0.4 and *χ*_AB_ = 0 [and at *R*_d_/*a* = 20 for the pair of *ϕ*_P,d_(*R*_d_) and *ϕ*_P,h_(*R*_d_)]. Figure 3 shows the phase diagram under this condition. Here, the black, blue, and red curves indicate coexisting homogeneous, droplet, and micellar phases, respectively, and solid and broken curves the stable and unstable (or metastable) states, respectively. The small black circle in the figure indicates the critical point for the liquid–liquid phase separation. It was noted that there is no critical point for the homogeneous-droplet phase equilibrium. For the coexistence of the micellar and homogeneous phases, we can draw two common tangents (the two tangents become identical at *χ*_BS_ = 1.122 in Figure 2), and the phase diagram has two coexistence regions of the micelle + dilute phase and micelle + concentrated phase, as shown in Figure 3. In what follows, we focused only on micellization and the concentrated-phase droplet formation in dilute copolymer solutions.

The micellar phase formation thermodynamically prefers the formation of the droplet phase with *R*_d_/*a* = 20 at *χ*_BS_ > 1.048, as indicated by the blue thin dotted line in Figure 3. This phase boundary shifts to lower *χ*_BS_ than the boundary between the micellization and macroscopic liquid–liquid phase separation (cf. the red thin dotted line in Figure 3), because the interfacial tension term in ∆*g*_d_(*ϕ*_P_) given by Equation (7) destabilizes the droplet concentrated phase. With increasing *R*_d_, the boundary between the micelle and droplet phases approaches the red thin dotted line, located at *χ*_BS_ = 1.122.

As already shown in a previous work [27], when *χ*_AS_ decreases, the biphasic region of the liquid–liquid phase separation goes up (Figure 3), while the binodal curves for the micelle–homogeneous phase equilibrium do not change as much. Thus, the stable liquid–liquid phase separation region becomes narrower and finally disappears in the phase diagram. A similar change in the phase diagram occurs when *χ*_AB_ increases.

In Figure 3, the black thin dot-dash curve indicates the spinodal, calculated by [29]

(15) $$\phi_{P,\mathrm{sp}\pm }=\frac{2\bar{\chi }-1+P^{-1}\pm \sqrt{{\left(2\bar{\chi }-1+P^{-1}\right)}^{2}-8P^{-1}\bar{\chi }}}{4\bar{\chi }}$$

within the spinodal region, the homogeneous phase is not stable with respect to long-ranged concentration fluctuation induced by thermal agitation, and the spinodal decomposition can take place in the early stage of the liquid–liquid phase separation. On the other hand, the concentration fluctuation is unstable in the homogeneous solution, outside the spinodal region. Thus, when the homogeneous solution is jumped into the region between the spinodal and binodal curves in the dilute region, the concentrated-phase droplet and spherical micelle may be formed in the solution through the nucleation–growth mechanism.

## 3. Kinetics of Micellization and Liquid–Liquid Phase Separation

### 3.1. Growth of the Concentrated-Phase Droplet and Spherical Micelle in the Early Stage

Let us consider a metastable dilute homogeneous solution with a copolymer volume fraction *ϕ*_P,h_ between *ϕ*_P,h_(*R*_d_) and the lower spinodal volume fraction *ϕ*_P,sp−_ (cf. Equation (15)). When a spherical concentrated-phase droplet with the radius *R*_d_ and the copolymer volume fraction *ϕ*_P,d_ is formed in this homogeneous copolymer solution, the Gibbs energy of the droplet formation *δG*_d_ is given by

(16) $$\delta G_{d}=\frac{\Delta g_{d}(\phi_{P,d})-\Delta g_{h}(\phi_{P,h})}{\phi_{P,d}}\left(P_{A}+P_{B}\right)m$$

where *ϕ*_P,d_ is assumed to be equal to the equilibrium copolymer volume fraction *ϕ*_P,d_(*R*_d_) calculated by the phase equilibrium condition, Equations (13a) and (13b), as a function of the droplet radius *R*_d_, and the aggregation number *m* is calculated by

(17) $$m=\frac{4\pi }{3\left(P_{A}+P_{B}\right)}R_{d}{}^{3}\phi_{P,d}$$

Because the droplet of the radius *R*_d_ is in equilibrium with the homogeneous solution with *ϕ*_P,h_(*R*_d_) (<*ϕ*_P,h_), there was a concentration gradient in the vicinity of the droplet surface and the droplet grew through the diffusion flow of the copolymer from the homogeneous solution.

Similarly, when a spherical micelle with the aggregation number *m* and the copolymer volume fraction *ϕ*_P,m_ formed in the dilute homogeneous copolymer solution with the copolymer volume fraction *ϕ*_P,h_ (*ϕ*_P,h_^(m)^ < *ϕ*_P,h_ < *ϕ*_P,sp−_), the Gibbs energy of the micelle formation *δG*_m_ is given by

(18) $$\delta G_{m}=\frac{\Delta g_{m}(\phi_{P,m})-\Delta g_{h}(\phi_{P,h})}{\phi_{P,m}}\left(P_{A}+P_{B}\right)m$$

where *ϕ*_P,m_ is calculated from *m* by

(19) $$\phi_{P,m}=\frac{a^{3}\left(P_{A}+P_{B}\right)}{\left(4\pi /3\right)R_{m}{}^{3}}m$$

where *R*_m_ is calculated by Equation (1).

**Figure 4:**
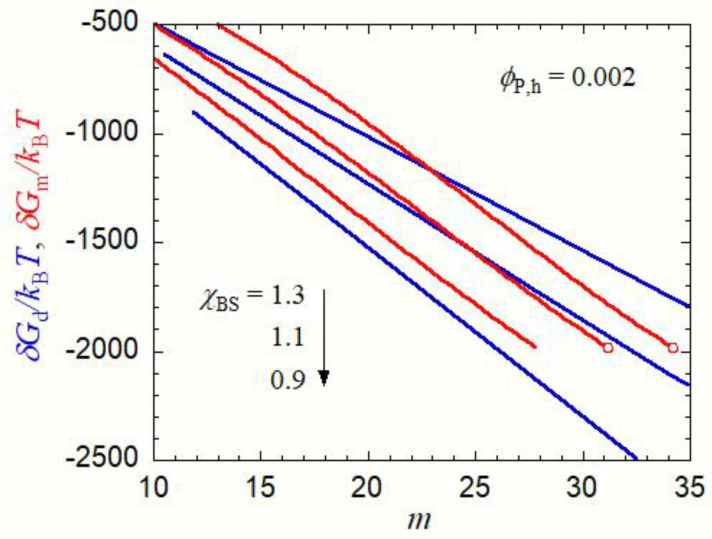
Gibbs energy of the droplet formation *δG*_d_ (blue curves) and the Gibbs energy of the micelle formation *δG*_m_ (red curves) in the copolymer solution with *P*_A_ = *P*_B_ = 100, *χ*_AS_ = 0.4, *χ*_AB_ = 0, and *χ*_BS_ = 1.3, 1.1, and 0.9 at *ϕ*_P,h_ = 0.002, as functions of the aggregation number *m*. Red circles indicate the points corresponding to the equilibrium micelle, fulfilling Equations (13a) and (13b).

In Figure 4, *δG*_d_ and *δG*_m_ are plotted against the aggregation number *m* in the copolymer solution at *ϕ*_P,h_ = 0.002. As seen in Figure 3, micellization is preferred to the liquid–liquid phase separation at *χ*_BS_ = 1.3, but *δG*_m_ for the micelle is higher than *δG*_d_ for the concentrated-phase droplet at *m* < 23. Thus, if *χ*_BS_ abruptly changes from a low value (<0.85, the one-phase condition) to 1.3, small concentrated-phase droplets form more easily than the micelles with the same *m* (<23) in the copolymer solution. However, when these concentrated-phase droplets grow, they become less stable than the micelle at *m* > 23. This indicates that the core–shell structure may be developed inside the growing droplets. Finally, the micelles with *m* = 34 (the red circle in Figure 4 for *χ*_BS_ = 1.3) form in the copolymer solution in the equilibrium state.

A similar relation between *δG*_d_ and *δG*_m_ also holds at *χ*_BS_ = 1.1. Concentrated-phase droplets first form in the copolymer solution, and they may be transformed into micelles with *m* = 31 (the red circle in Figure 4 for *χ*_BS_ = 1.1) during the growing process. Although the liquid–liquid phase separation is preferred to the micellization at *χ*_BS_ = 1.1 in the final equilibrium state, as seen in Figure 3, the micelle is a metastable state, and the formation of the concentrated phase of a large size must overcome the activation Gibbs energy barrier. The late stage of the concentrated-phase droplet growth in such a solution is discussed in the next Section 3.2.

At *χ*_BS_ = 0.9, *δG*_d_ is smaller than *δG*_m_ in the whole *m* range, and the concentrated-phase droplet keeps growing without transforming into the micelle. In the equilibrium state, the liquid–liquid phase separation is preferred to micellization (cf. Figure 3).

The kinetics of liquid–liquid phase separation and micellization in the early stage has been argued in the scenario of the nucleation–growth mechanism thus far. That is, the nuclei of the concentrated phase and the micelle are formed step-wise from the unimer (*m* = 1) [17,18,19,20,21]. In the concentrated-phase droplet, the copolymer chain was assumed to take the random coil conformation, and the concentration gradient near the droplet surface was neglected to derive Equation (7). Thus, Equation (7) for ∆*g*_d_(*ϕ*_P_) holds only at *R*_d_ larger than <*R*^2^>^1/2^/2 + *d*_I,d_, where <*R*^2^>^1/2^ is the mean-square end-to-end distance of the copolymer chain [=(*P*_A_ + *P*_B_)*a*^2^], and *d*_I,d_ is calculated by Equation (9). In Figure 4, *m* = 10 is close to the lower limit where Equation (7) holds. Moreover, in the micelle model used in this work, *R*_m_ and *R*_core_ are given by Equation (1), which may hold only when *ϕ*_B,c_ is sufficiently high. For small *m*, where *ϕ*_B,c_ is low, B-block chains forming the core may shrink to escape contact with the solvent, so our micelle model cannot be used for such a small *m*. Under the condition of Figure 4, *ϕ*_B,c_ ≈ 0.25 at *m* = 10, and our micelle model may not be applied at *m* considerably smaller than 10. Therefore, Equations (16) and (18) cannot be used to discuss the nucleation processes of liquid–liquid phase separation and micellization [21].

Although these nucleation processes were not discussed, the above argument proposes a new kinetics of the micellization process in the early stage (i.e., the transformation from the small concentrated-phase droplet to the spherical micelle). As above-mentioned, the stable liquid–liquid phase separation region becomes narrower and finally disappears in the phase diagram, when *χ*_AS_ decreases and/or *χ*_AB_ increases. Nevertheless, the same kinetics of the micellization process in the early stage can also be expected at lower *χ*_AS_ and higher *χ*_AB_, because *δG*_d_ < *δG*_m_ at small *m*, even if the liquid–liquid phase separation region becomes unstable, as demonstrated in Figure 4 at *χ*_BS_ = 1.3.

### 3.2. Modification of the Lifshitz–Slyosov Theory in the Late Stage of the Phase Separation

As above-mentioned, the micellar phase is the metastable state at *χ*_BS_ = 1.1 (more generally at *χ*_BS_ from 1.03 to 1.122) within the biphasic region in Figure 3, and the thermodynamically equilibrium state is the liquid–liquid phase separation (where the concentrated-phase droplet size is macroscopic) in that region in Figure 3. The phase boundary (the blue horizontal dotted line in Figure 3 at *R*_d_/*a* = 20) shifts to *χ*_BS_ = 1.1 at *R*_d_/*a* = 50, so the concentrated-phase droplet with *R*_d_/*a* > 50 is stabler than the micelle. If the concentrated-phase droplet of such a large size is formed occasionally by overcoming the activation Gibbs energy barrier, it keeps growing without developing the micellar structure. Since *ϕ*_P,h_(*R*_d_) < *ϕ*_P,h_^(m)^ at *R*_d_/*a* > 50, the growth of such large droplets in the solution is accompanied by the dissociation of micelles existing in the solution. This droplet ripening process in the micellar solution is different from the conventional Ostwald ripening in the usual liquid–liquid phase separation of the late stage.

The conventional Ostwald ripening process is quantitatively dealt with by the Lifshitz–Slyozov theory [31,32]. In the theory, the droplet with the radius *R*_d_ is in equilibrium with the homogeneous dilute solution (the mother solution) with the concentration *ϕ*_P,h_(*R*_d_) given by (in terms of our notations)

(20) $$\phi_{P,h}(R_{d})=\phi_{P,h}(\infty )+\frac{\sigma }{R_{d}}$$

where *σ* is a constant parameter proportional to the interfacial tension. When the average concentration of the mother solution is denoted as ${\bar{\phi }}_{P,h}$, larger droplets with *ϕ*_P,h_(*R*_d_) < ${\bar{\phi }}_{P,h}$ can grow, while smaller droplets with *ϕ*_P,h_(*R*_d_) > ${\bar{\phi }}_{P,h}$ dissociate along with diffusing the solute into the mother solution. The critical radius R* for the growing droplet is given by

(21) $$R^{*}=\frac{\sigma }{{\bar{\phi }}_{P,h}-\phi_{P,h}(\infty )}$$

Along with the droplet growth, the concentration of the mother solution is reduced, and R* increases with time *t*. Although the *σ* calculated from the results of *ϕ*_P,h_(*R*_d_) obtained by Equations (13a) and (13b) was slightly dependent on *R*_d_, we neglected this dependence according to Lifshitz and Slyozov in what follows, of which the approximation is good only at sufficiently large *R*_d_, or in the late stage of the phase separation

The concentration of the mother solution containing spherical micelles should maintain the critical micelle concentration *ϕ*_P,h_^(m)^, even during the growth of concentrated-phase droplets. Thus, Equation (21) should be replaced by

(22) $$R^{*}=\frac{\sigma }{\phi_{P,h}{}^{\left(m\right)}-\phi_{P,h}(\infty )}$$

Thus, R* is independent of *t* until all micelles disappear in the solution, which is in contrast with the Lifshitz–Slyozov theory, and the theory should be modified as follows.

New parameters *u* and *t*_r_ are defined by

(23) $$u\equiv \frac{R_{d}}{R^{*}},t_{r}\equiv \frac{\sigma D}{R^{*}{}^{3}}t$$

where *D* is the diffusion coefficient of the solute in the mother solution. Using these parameters, the droplet growth rate d*u*/d*t*_r_ can be written as

(24) $$\frac{du}{dt_{r}}=\frac{1}{u}\left(1-\frac{1}{u}\right)$$

**Figure 5:**
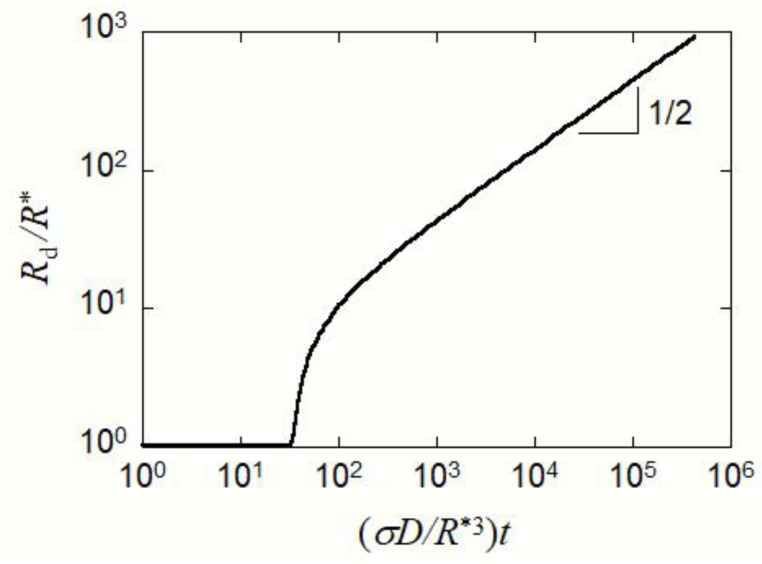
Time dependence of the growing droplet radius (*R*_d_ = R* at *t* = 0) in the micellar solution, calculated by Equation (25).

By integrating this equation, we obtain

(25) $$\frac{1}{2}\left(u+3\right)(u-1)+\ln(u-1)+C_{0}=t_{r}$$

with the integration constant *C*_0_. Figure 5 shows the time dependence of *R*_d_ for the growing droplet (*R*_d_ = R* at *t* = 0) in the micellar solution, calculated by Equation (25). When *u* and *t*_r_ are sufficiently large, Equation (25) can be approximated to

(26) $$u\approx \sqrt{2t_{r}}$$

This is in contrast with the Lifshitz–Slyozov theory, where the average radius of the droplet is proportional to *t*^1/3^ in the late stage [31,32]. After all micelles disappear in the solution, the droplet growth should obey the Ostwald ripening (i.e., larger droplets grow by consuming the solute provided by dissociating smaller droplets). As the result, the average radius of the growing droplet is proportional to *t*^1/3^.

## 4. Concluding Remarks

A discussion has been made on the phase separation kinetics in the block copolymer solution where the micellization competes with the liquid–liquid phase separation. When the block copolymer solution is jumped from the one-phase to biphasic region near the micelle–liquid–liquid phase separation boundary, small droplets of the concentrated phase are first formed, and afterward, the micellar structure may be developed in the concentrated-phase droplets. This is a kinetic mechanism of the micellization newly proposed in this study.

The kinetics of the micelle formation in block copolymer solutions has been investigated experimentally by many researchers by using the temperature-jump and stopped flow experiments [1,2,3,4,5,6,7,8,9,10,11,12,13,14,15,16]. When homogeneous block copolymer solutions were brought into the micelle region, the scattering intensity and hydrodynamic radius in the copolymer solutions increased with time, and these experimental results were analyzed in terms of the step-by-step association model [17,18,19,20,21], but the detailed structure inside the scattering particles have not been investigated thus far. It is not enough to only measure the scattering intensity and hydrodynamic radius to distinguish the micelle from the concentrated-phase droplet experimentally. The newly proposed micellization kinetics must be checked in future work.

According to the above micellization kinetics, the micelles exist as a metastable state, even out of the micellization region in the thermodynamically stable phase diagram. In the late stage of the phase separation, the metastable micelle may be transformed into the stabler concentrated phase through the ripening process of the concentrated-phase droplet. This ripening process differs from the conventional Ostwald ripening occurring in the usual liquid–liquid phase separation systems [31,32], because the droplet growth in our block copolymer solution takes place by consuming the solute provided by metastable micelles in the solution. The growing droplet size is proportional to the square root of time *t* in the micellar solution, and the conventional *t*^1/3^ dependence of the (average) droplet size in the Ostwald ripening is recovered after all micelles are consumed.

Recently, Narang and Sato [33] investigated the self-assembly of an amphiphilic amino acid derivative in aqueous dimethylsulfoxide, where concentrated-phase droplets coexist with micellar particles. Unfortunately, after the temperature jump of the solution, light scattering indicated the existence of sub-micron size droplets, but the droplets did not essentially grow any more during the light scattering and small-angle X-ray scattering measurements, maybe because of the fast ripening process in this small molecular system. To the best of the author’s knowledge, there have been no reports demonstrating the *t*^1/2^ dependence of the droplet size in phase separating solutions.

## Appendix A. Interfacial Tension and Interfacial Thickness

Noolandi and Hong [30] formulated the interfacial tension *γ* and interfacial thickness *d*_I_ for phase separating solutions containing two homopolymers A and B and a solvent S. They simply assumed linear concentration gradients in the interfacial region shown in Figure A1. Here, *x* is the distance from the middle of the interface, *ϕ*_A_(*x*), *ϕ*_B_(*x*), and *ϕ*_S_(*x*) [=1 − *ϕ*_A_(*x*) − *ϕ*_B_(*x*)] are the volume fractions of the A and B homopolymers and the solvent, respectively, at *x*; *ϕ*_A_^(*α*)^, *ϕ*_B_^(*α*)^, and *ϕ*_S_^(*α*)^ are *ϕ*_A_(*x*), *ϕ*_B_(*x*), and *ϕ*_S_(*x*) at *x* ≤ −*d*_I_/2 (the *α* or homopolymer A rich bulk phase), and *ϕ*_A_^(*β*)^, *ϕ*_B_^(*β*)^, and *ϕ*_S_^(*β*)^ are *ϕ*_A_(*x*), *ϕ*_B_(*x*), and *ϕ*_S_(*x*) at *x* ≥ *d*_I_/2 (the *β* or homopolymer B rich bulk phase).

Their results of the interfacial tension *γ* and interfacial thickness *d*_I_ are written as

(A1) $$\frac{a^{2}\gamma }{k_{B}T}=\sqrt{\frac{1}{3}K_{\mathrm{AB}}\Delta f_{\mathrm{AB}}(0)},\frac{d_{I}}{a}=\sqrt{\frac{K_{\mathrm{AB}}}{12\Delta f_{\mathrm{AB}}(0)}}$$

where *a* is the unit lattice size; *k*_B_*T* is the Boltzmann constant multiplied by the absolute temperature; and the parameters *K*_AB_ and ∆*f*_AB_(0) are defined by

(A2) $$K_{\mathrm{AB}}\equiv \frac{{\left(\phi_{A}{}^{\left(\beta \right)}-\phi_{A}{}^{\left(\alpha \right)}\right)}^{2}}{\phi_{A}{}^{\left(\beta \right)}+\phi_{A}{}^{\left(\alpha \right)}}+\frac{{\left(\phi_{B}{}^{\left(\beta \right)}-\phi_{B}{}^{\left(\alpha \right)}\right)}^{2}}{\phi_{B}{}^{\left(\beta \right)}+\phi_{B}{}^{\left(\alpha \right)}}$$

(A3) $$\begin{array}{cc} 4\Delta f_{\mathrm{AB}}(0) & \equiv \chi_{\mathrm{AB}}\left(\phi_{A}{}^{\left(\alpha \right)}-\phi_{A}{}^{\left(\beta \right)}\right)\left(\phi_{B}{}^{\left(\beta \right)}-\phi_{B}{}^{\left(\alpha \right)}\right)\\ & +\left[\chi_{\mathrm{AS}}\left(\phi_{A}{}^{\left(\alpha \right)}-\phi_{A}{}^{\left(\beta \right)}\right)-\chi_{\mathrm{BS}}\left(\phi_{B}{}^{\left(\beta \right)}-\phi_{B}{}^{\left(\alpha \right)}\right)\right]\left(\phi_{S}{}^{\left(\beta \right)}-\phi_{S}{}^{\left(\alpha \right)}\right)\\ & +2\phi_{S}{}^{\left(\beta \right)}\ln\left(\frac{\phi_{S}{}^{\left(\beta \right)}+\phi_{S}{}^{\left(\alpha \right)}}{2\phi_{S}{}^{\left(\beta \right)}}\right)+2\phi_{S}{}^{\left(\alpha \right)}\ln\left(\frac{\phi_{S}{}^{\left(\beta \right)}+\phi_{S}{}^{\left(\alpha \right)}}{2\phi_{S}{}^{\left(\alpha \right)}}\right) \end{array}$$

For phase separating binary solutions containing a homopolymer P and a solvent S, the above equations can be simplified by

(A4) $$\frac{a^{2}\gamma }{k_{B}T}=\sqrt{\frac{1}{3}K_{P}\Delta f_{P}(0)},\frac{d_{I}}{a}=\sqrt{\frac{K_{P}}{12\Delta f_{P}(0)}}$$

(A5) $$K_{P}\equiv \frac{{\left(\phi_{P}{}^{\left(\beta \right)}-\phi_{P}{}^{\left(\alpha \right)}\right)}^{2}}{\phi_{P}{}^{\left(\beta \right)}+\phi_{P}{}^{\left(\alpha \right)}}$$

(A6) $$\begin{array}{cc} 4\Delta f_{P}(0) & \equiv \chi \left(\phi_{P}{}^{\left(\beta \right)}-\phi_{P}{}^{\left(\alpha \right)}\right)\left(\phi_{S}{}^{\left(\alpha \right)}-\phi_{S}{}^{\left(\beta \right)}\right)\\ & +2\phi_{S}{}^{\left(\beta \right)}\ln\left(\frac{\phi_{S}{}^{\left(\beta \right)}+\phi_{S}{}^{\left(\alpha \right)}}{2\phi_{S}{}^{\left(\beta \right)}}\right)+2\phi_{S}{}^{\left(\alpha \right)}\ln\left(\frac{\phi_{S}{}^{\left(\beta \right)}+\phi_{S}{}^{\left(\alpha \right)}}{2\phi_{S}{}^{\left(\alpha \right)}}\right) \end{array}$$

where *ϕ*_P_^(*α*)^ and *ϕ*_P_^(*β*)^ are the polymer volume fractions in the *α* phase (dilute phase) and the *β* phase (concentrated phase), respectively.
